# Oxygen Vacancy Ordering and Molten Salt Corrosion Behavior of ZnO-Doped CeYSZ for Solid Oxide Membranes

**DOI:** 10.3390/nano13202790

**Published:** 2023-10-18

**Authors:** Hwanseok Lee, Heesoo Lee

**Affiliations:** Department of Materials Science and Engineering, Pusan National University, Busan 46241, Republic of Korea; hwanseok@pusan.ac.kr

**Keywords:** solid oxide electrolyte, local atomic structure, destabilization, corrosion resistance, yttrium depletion layer

## Abstract

Although 4Ce4YSZ has high corrosion resistance, it faces challenges concerning its sinterability and ionic conductivity. Therefore, we studied destabilization behavior caused by corrosion and oxygen vacancy ordering according to ZnO doping. Powders of (4Ce4YSZ)_1−x_(ZnO)_x_ (x = 0.5, 1, 2, 4 mol%) were synthesized using the sol-gel method. With the addition of ZnO, the cubic phase increased, and secondary phases were not observed. The (111) peak showed a higher angle shift in ZnO-doped 4Ce4YSZ compared to 4Ce4YSZ, and TEM-SAED revealed a reduction in the spacing of the (011)t plane, suggesting lattice contraction due to the substitution of the smaller Zn^2+^ (60 Å) for Zr^4+^ (84 Å) in the lattice. The local atomic structure analysis was conducted using EXAFS to investigate the oxygen vacancy ordering behavior. Zr K-edge Fourier transform data revealed a decrease in the Zr-O1 peak intensity with an increasing amount of ZnO doping, indicating an increase in oxygen vacancies. The Zr-O1 peak position shifted to the right, leading to an increase in the Zr-O1 interatomic distance. In the Y K-edge Fourier transform data, the Y-O1 peak intensity did not decrease, and there was little variation in the Y-O1 interatomic distance. These results suggest that the oxygen vacancies formed due to ZnO doping are located in the neighboring oxygen shell of Zn, rather than in the neighboring oxygen shells of Y and Zr. Impedance measurements were conducted to measure the conductivity, and as the amount of ZnO doping increased, the total conductivity increased, while the activation energy decreased. The increase in oxygen vacancies by ZnO doping contributed to the enhancement of conductivity, and it is considered that these created oxygen vacancies did not interact with Zn^2+^ and did not form defect associations. Fluoride-based molten salts were introduced to the specimens to assess the corrosion behavior in a molten salt environment. Yttrium depletion layers (YDLs) were formed on the surfaces of all specimens due to the leaching of yttrium. However, Ce remained relatively stable at the interface according to EDS line scans, suggesting a reduction in the phase transformation (cubic, tetragonal to monoclinic) typically associated with yttrium leaching in YSZ.

## 1. Introduction

The solid oxide membrane (SOM) process has attracted considerable attention as a new metal reduction technology due to its high cost-effectiveness and low greenhouse gas emissions [1]. Yttria-stabilized zirconia (YSZ) is the preferred material for SOM due to its high oxygen ion conductivity within the operating temperature range (1000–1300 °C) [2]. However, corrosion issues with YSZ in molten fluoride flux have become a limiting factor in the operational lifespan of SOM electrolysis processes.

Studies are underway to enhance molten salt corrosion resistance caused by molten fluoride flux, as it is crucial for the solid oxide membrane (SOM) process. Various methods have been investigated to prevent yttrium leaching in molten electrolytes, including the addition of additives such as YF_3_, Y_2_O_3_, and MgO. However, these are not suitable alternatives because they can inevitably influence the electrolyte [3,4,5,6]. Research has been conducted to investigate new compositions or additives to address the issue of yttrium leaching. Calcia-stabilized zirconia (CSZ) exhibits lower ionic conductivity and corrosion resistance compared to YSZ, while magnesia-stabilized zirconia (MSZ) demonstrates superior corrosion resistance to YSZ but has a higher tendency for spontaneous destabilization at operating temperatures (1000–1300 °C) and lower ionic conductivity [7,8,9,10]. As a result, research has been conducted to improve the corrosion resistance by doping more acidic stabilizing elements (such as CeO_2_, In_2_O_3_, Sc_2_O_3_, etc.) into YSZ. It has been observed that CeO_2_ co-doped yttria-stabilized zirconia exhibits enhanced corrosion resistance [11,12]. However, an increase in the amount of CeO_2_ doping has been found to lead to a decrease in the cubic phase and ionic conductivity, along with the drawback of poor sinterability [13].

Zirconia is stabilized into high-temperature tetragonal and cubic phases by adding lower-valency cations. The doped ions create oxygen vacancies to maintain electrical neutrality. Ion conduction in zirconia occurs through thermally activated hopping, involving the long-range transport of oxygen ions to the nearest oxygen vacancies [14]. The location of the oxygen vacancy is very important because the oxygen ion conductivity in zirconia is highly dependent on the structure around the oxygen vacancies [15]. While simple electrostatic arguments suggest that oxygen vacancies are positioned near the doped cations, the actual placement is known to vary depending on the valence state and ion size of the dopants. Theoretically, density-functional theory (DFT) modeling can be used to calculate the concentration and positions of oxygen vacancies, and this is being investigated for its role in studying the oxygen ion transport mechanism [16]. The structural changes induced by oxygen vacancies can be analyzed using X-ray diffraction (XRD) and neutron powder diffraction (NPD). NPD is easily detectable through neutron powder diffraction due to the relatively large neutron scattering length of oxygen atoms (5.805 ± 0.004 fm) [17]. Neutron diffraction is a method that can determine the crystallographic positions of oxygen ions, along with their atomic displacement parameters and occupancies [18]. NPD is used to analyze crystalline materials by exploiting the structural periodicity, and diffraction by the crystal lattice emphasizes a long-range order, allowing it to obscure diffused scattering caused by disorder. Extended X-ray absorption fine structure (EXAFS) spectroscopy is suited for the investigation of the local structure of disordered materials because the photoelectron probes specifically around the absorbing atom [19]. Specifically, EXAFS measures vibrations observed on the high-energy side of the X-ray absorption edge. The amplitude and frequency of these vibrations vary depending on the number, types, and distances of neighboring atoms around the atoms involved in the X-ray absorption [20]. Therefore, utilizing X-ray absorption fine structure spectroscopy (XAFS) to analyze the local atomic structure around specific atoms is an effective approach [21,22,23]. Among dopants, ZnO is expected to not only enhance phase stability but to also serve as a sintering aid. However, there has been no research on the formation and ordering behavior of oxygen vacancies due to ZnO doping.

We studied the formation and ordering of oxygen vacancies and the destabilization behavior due to corrosion in 4Ce4YSZ with ZnO doping. XRD analysis was performed to confirm the phase transformation resulting from ZnO doping, and interplanar distances were measured through TEM-SAED analysis. The formation and ordering behavior of oxygen vacancies were analyzed using extended X-ray absorption fine structure (EXAFS), and the conductivity was investigated through impedance spectroscopy. A molten salt test was conducted to confirm the corrosion resistance of ZnO-doped 4Ce4YSZ, and the resulting destabilization behavior was analyzed.

## 2. Experiment

Using the sol-gel method, 4Ce4YSZ powders were synthesized [24]. ZrCl_4_ (ATI, Leawood, KS, USA, ≥99.9%), Y(NO_3_)_3_·6H_2_O (Sigma-Aldrich, St. Louis, MO, USA, ≥99.8%), and Ce(NO_3_)_3_·6H_2_O (Alfa Aesar, Haverhill, MA, USA, ≥99.9%) precursors were dissolved in deionized water according to the stoichiometry of the composition to prepare a homogeneous mixed-metal solution. Then, the cation solutions, citric acid (CA) and ethylene glycol (EG), were mixed in a beaker, which was called a sol state, in sequence at a total metal ion/citric acid/ethylene glycol mole ratio of 1:4:16. The sol was heated with stirring at 80 °C, and the pH was adjusted to approximately 10 by adding a 1 N NH_4_OH solution. An opaque viscous gel obtained by continuous stirring and heating was baked to solidify in an oven at 400 °C for 4 h. The solidified precursors were then calcinated in air at 1200 °C for 2 h. The powder was uniaxially pressed at 3 ton/m^2^ to produce a 20 mm disk specimen, and the green body was sintered at 5 °C/min to various temperatures (1200–1600 °C) for 2 h. ZnO (Sigma-Aldrich, ≥99.99%) was introduced to the 4Ce4YSZ powders by the mechanical mixing method. The mixed powders were ball-milled in ethanol for 24 h and subsequently dried. The dried powders were calcinated in air at 1200 °C.

A molten salt test was conducted using the eutectic composition of calcium fluoride (98%, Junsei Chemical Co., Ltd., Chuo-ku, Tokyo, Japan) and sodium fluoride (98%, Junsei Chemical Co., Ltd.) by ball-milling at 200 rpm in Nalgene bottles for 12 h. Then, typically, 0.7 g of the mixed powder was pelletized (1 ton/m^2^) in a cylindrical 15 mm die. The obtained fluoride composite green bodies were then attached to the surfaces of discs and heated at 1000 °C for 100 h. 

The densities of the sintered pellets were determined using the Archimedes method by immersing the samples in distilled water. X-ray diffraction (XRD) patterns of the specimens were collected at room temperature using a step scan procedure (2θ = 10–90°, with a step interval of 0.02°) and Cu-Kα radiation on a Rigaku Ultima-IV XRD instrument (Rigaku Corporation, Akishima-shi, Tokyo, Japan). Transmission electron microscopy (TEM, JEOL, JEM-2100) (Nanoscience Instruments, Phoenix, AZ, USA) was used at the KBSI Busan center to analyze the microstructure of the powders. Extended X-ray absorption fine structure spectroscopy (EXAFS) experiments were conducted at both the Zr K-edge and Y K-edge using the EXAFS facility at the 7D XAFS beamline in the Pohang Accelerator Laboratory (PLS-II, Pohang, Republic of Korea). AC impedance measurements were carried out using an Ivium-Stat instrument (Ivium, Eindhoven, The Netherlands) within a frequency range from 10^6^ Hz to 10^−2^ Hz, with an excitation voltage of 10 mV, at an operating temperature of 700 °C, under air conditions. The SEM images of the samples were obtained by using a JSM-IT800 scanning electron microscope.

## 3. Results and Discussion 

**Figure 1:**
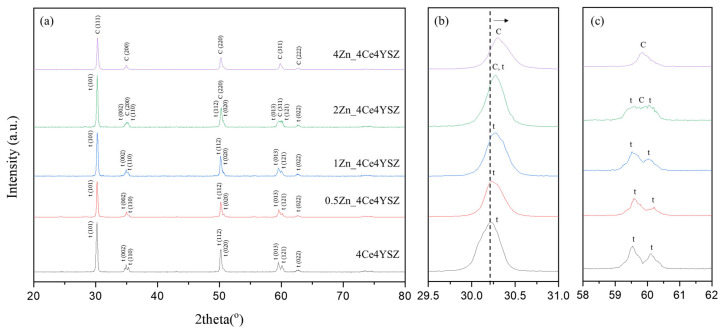
(**a**) XRD diffraction patterns, (**b**) (111) peaks, and (**c**) (311) peaks of ZnO-doped 4Ce4YSZe.

The XRD patterns of the powders calcined in air at 1200 °C for 2 h are shown in Figure 1. It confirms the formation of the tetragonal phase (PDF 49-1642) in 4Ce4YSZ. With an increase in the amount of ZnO doping, the cubic phase (PDF 50-1089) gradually became more prominent, as shown in Figure 1c. In 4Zn_Ce4YSZ, the cubic phase was dominant, and no other phases were observed. As shown in Figure 1b, with the increasing concentrations of ZnO, the (111) peak shifted to a higher angle. This result suggests that Zn^2+^ ions, with a smaller ionic radius (60 Å) than Zr^4+^ (84 Å), are incorporated into the solid solution as substitutional elements, stabilizing the cubic phase through the formation of oxygen vacancies [25].

**Figure 2:**
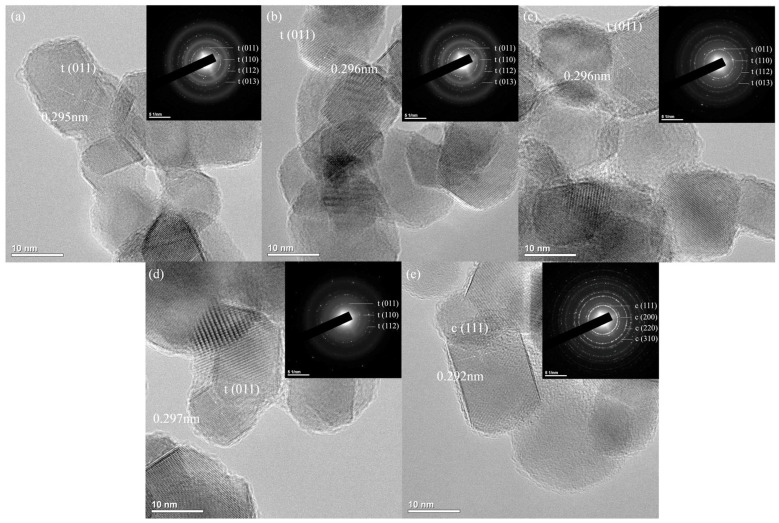
TEM images and SAED patterns of (**a**) 4Ce4YSZ, (**b**) 0.5Zn_4Ce4YSZ, (**c**) 1Zn_4Ce4YSZ, (**d**) 2Zn_4Ce4YSZ and (**e**) 4Zn_4Ce4YSZ.

TEM images of Zn-doped 4Ce4YSZ are shown in Figure 2. The powders synthesized using the sol-gel method had an average size of 20–30 nm, and no significant changes in powder size were observed with increasing Zn doping levels. Tetragonal phases were confirmed in 4Ce4YSZ and 2Zn_4Ce4YSZ, while a cubic phase was observed in 4Zn_4Ce4YSZ. The lattice parameter tended to decrease as Zn doping increased through the d-spacing (0.296 nm, 0.293 nm) of 4Ce4YSZ and 2Zn_4Ce4YSZ, which was consistent with the XRD results.

**Figure 3:**
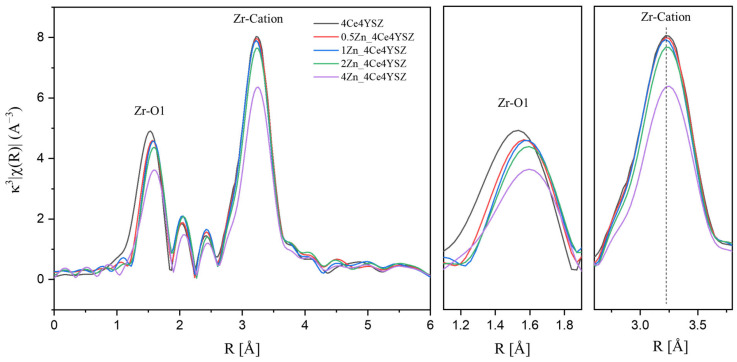
Zr K-edge Fourier transform data of ZnO-doped 4Ce4YSZ.

To investigate the formation and ordering behavior of oxygen vacancies due to Zn doping, Zr K-edge Fourier transform extended X-ray absorption fine structure (EXAFS) was conducted on powders calcinated in air at 1200 °C for 2 h. The results are presented in Figure 3, and the EXAFS signals were obtained in the range of 3 < K < 12 Å using a Hanning window. The first peak, appearing at approximately 1.5 Å, is attributed to the Zr-O bonding, while the second peak observed near 3.25 Å is associated with Zr-cation interactions [26,27]. The intensity of the Zr-O peak decreased with increasing ZnO doping. This result is attributed to the metallic substitution reaction, which forms oxygen vacancies in zirconia to maintain electron neutrality [28].

(1) $$ZnO\overset{ZrO_{2}}{\to }{Zn}_{zr}^{\prime \prime }+O_{O}^{X}+V_{O}^{••}$$

The interatomic distance between Zr and its first nearest neighbors showed an increasing trend with the increasing ZnO doping level. In yttria-stabilized zirconia, the presence of oxygen vacancies near Zr resulted in a reduction in the Zr-O interatomic distance, causing the cubic zirconia to have a smaller Zr-O interatomic distance compared to the tetragonal zirconia [26,29]. Therefore, it is considered that oxygen vacancies were formed near Zn^2+^ (60 Å), which was smaller than Zr^4+^ (84 Å) upon ZnO doping. This resulted in a decrease in oxygen vacancies near the zirconia, leading to an increase in the Zr-O1 interatomic distance. Additionally, a decrease in the intensity of the Zr-cation peak was observed, which can be attributed to an increase in the structural distortion with an increasing dopant concentration [30]. 

**Figure 4:**
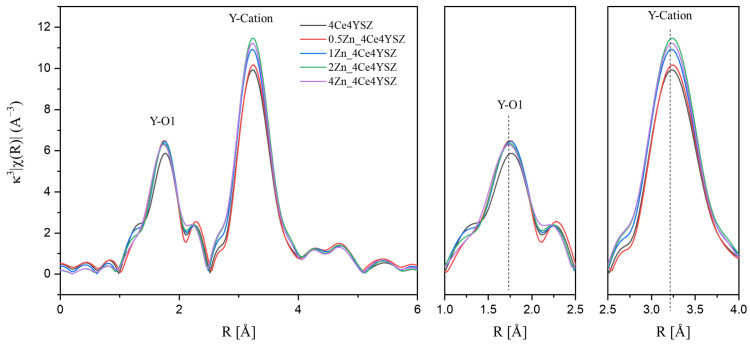
Y K-edge Fourier transform data of ZnO-doped 4Ce4YSZ.

Figure 4 displays the Y K-edge Fourier transform data, with the EXAFS signals obtained in the range of 3 < K < 11.5 Å using a Hanning window. The first peak, meaning Y-O bonding, appeared at approximately 1.75 Å, and the difference in the first cation–oxygen distances between Y-O and Zr-O is attributed to the different sizes of the Zr^4+^ and Y^3+^ ions [31]. The Y-O interatomic distance showed little variation, and there was almost no change in the intensity of the Y-O1 peak. These results indicate that the oxygen vacancies formed as a result of ZnO doping were not located in the Y neighboring oxygen shell. The reason the Y-O interatomic distance did not change, unlike the Zr-O interatomic distance, is because in 4Ce4YSZ, oxygen vacancies are primarily located in the Zr neighboring oxygen shell. It is presumed that the oxygen vacancies migrating upon ZnO doping are primarily located within the oxygen vacancies of the Zr neighboring oxygen shell, and it is concluded that there are no migrating oxygen vacancies within the Y neighboring oxygen shell.

**Figure 5:**
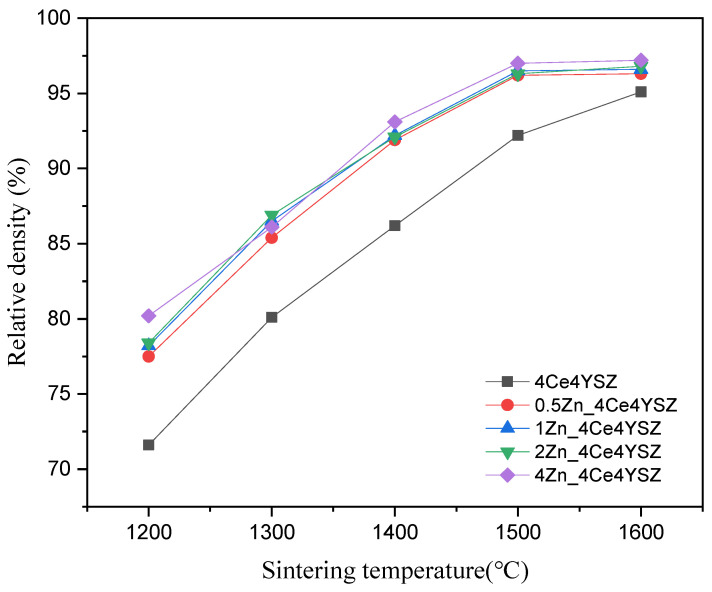
Relative density vs. sintering temperature for Zn-doped 4Ce4YSZ.

Figure 5 shows the calculated relative densities of the samples sintered at various temperatures with a duration of 2 h. As the sintering temperature increased, the relative density consistently increased, and 4Ce4YSZ increased from 71.6% (1200 °C) to 95.1% (1600 °C). On the other hand, 4Zn_4Ce4YSZ exhibited a relative density of 80.2% at 1200 °C, indicating a higher relative density than in the undoped case, and it reached a 97% relative density at 1500 °C. This confirms that Zn can address the sinterability issues associated with CeO_2_.

**Figure 6:**
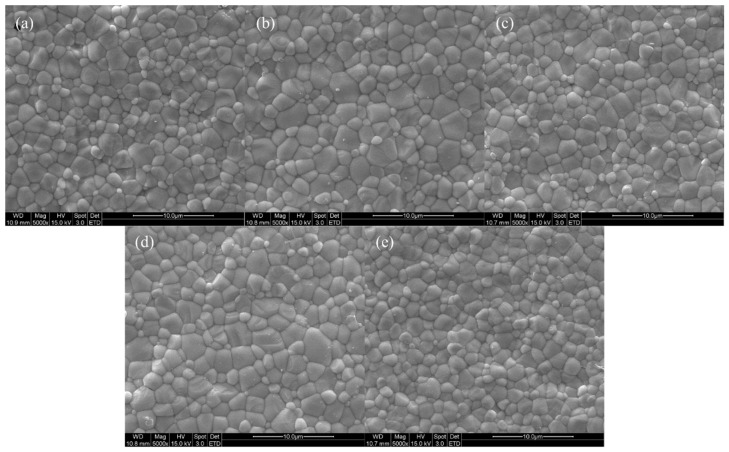
SEM surface images of (**a**) 4Ce4YSZ, (**b**) 0.5Zn_4Ce4YSZ, (**c**) 1Zn_4Ce4YSZ, (**d**) 2Zn_4Ce4YSZ and (**e**) 4Zn_4Ce4YSZ sintered at 1600 °C.

Figure 6 displays SEM images of the specimens sintered at 1600 °C for 2 h, confirming that all specimens were well sintered with a dense structure. The grain sizes were measured by analyzing the SEM images based on ISO 13383-1, resulting in grain sizes of 2.12, 2.17, 2.21, 2.30, and 2.16 μm, showing an increasing trend in grain size with Zn doping [32].

**Figure 7:**
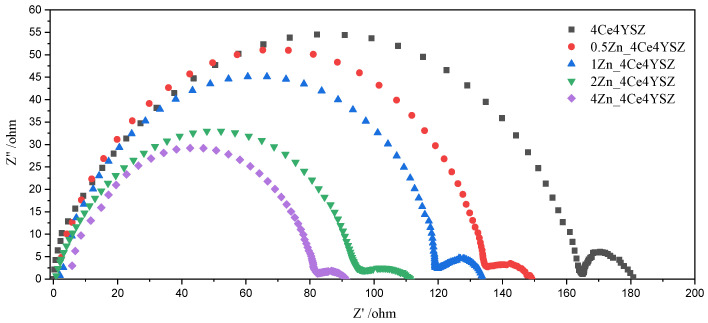
Nyquist plot of ZnO-doped 4Ce4YSZ obtained in air at 700 °C.

Figure 7 presents the Nyquist plot of impedance data measured at 700 °C. Two arcs represent the grain interior resistance (*R_gi_*) and the total grain boundary resistance (*R_gb_*), with the total resistance (*R_t_*) being the sum of these two arcs [33].

(2) $$R_{t}=R_{gi}+R_{gb}$$

**Figure 8:**
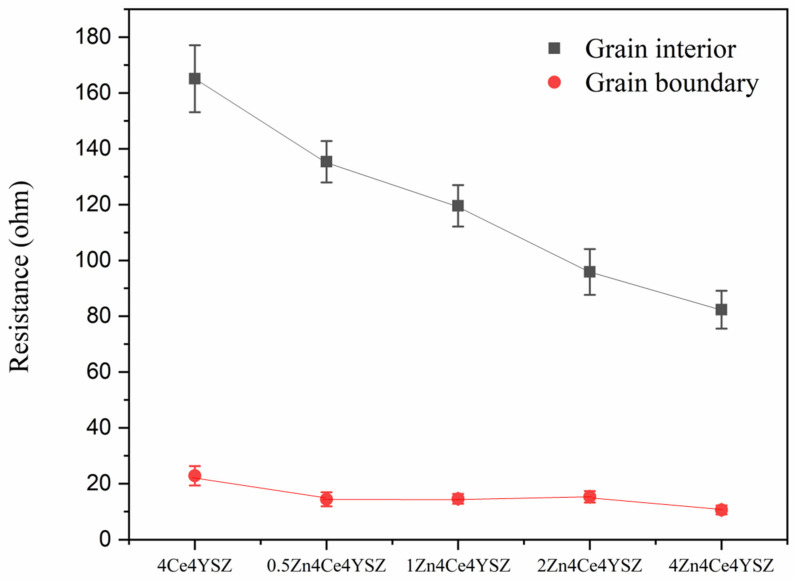
Resistances of grain interior and grain boundary derived from impedance spectroscopy.

In Figure 8, it was observed that *R_gi_* gradually decreased from 165.10 Ω (4Ce4YSZ) to 82.33 Ω (4Zn_4Ce4YSZ) with an increasing ZnO doping level. This result indicates that ZnO dissolved in ZrO_2_ forms oxygen vacancies, increasing the conductivity [28]. Similarly, *R_gb_* also gradually decreased from 22.84 Ω (4Ce4YSZ) to 10.60 Ω (4Zn_4Ce4YSZ). As the grain size increased with ZnO doping, the grain boundaries decreased. It is believed that ZnO could change the oxygen–ion conductive channel structure at the grain boundaries, leading to an improvement in *R_gb_* conductivity [34]. 

**Figure 9:**
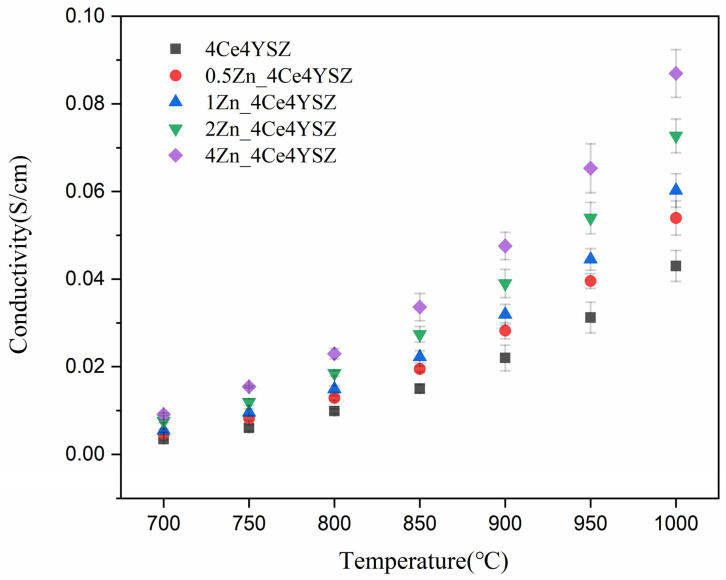
Total conductivity of ZnO-doped 4Ce4YSZ.

Figure 9 represents the total conductivity calculated based on impedance data and fitted with the Equation (3) below.

(3) $$\sigma_{t}=\frac{d}{SR_{t}}$$

where d represents the thickness of the specimen, while S is the area of the electrode. The conductivity, which was 0.0430 S/cm in 4Ce4YSZ, gradually increased with the increasing ZnO doping level, reaching 0.0870 S/cm in 4Zn_4Ce4YSZ. The increase in oxygen vacancies with increasing ZnO doping and the cubic phase formation contributed to the increase in conductivity [35]. 

**Figure 10:**
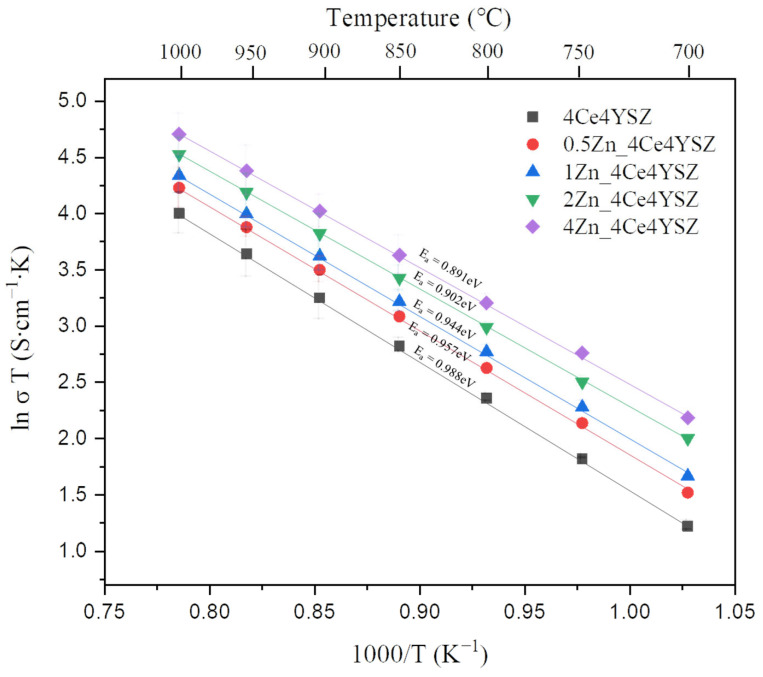
Arrhenius plots of the total conductivity in ZnO-doped 4Ce4YSZ.

Figure 10 represents the Arrhenius plots of total conductivity, and the dependence of ionic conductivity (σ) on temperature (T) was obtained using the Arrhenius equation.

(4) $$\sigma =\frac{\sigma_{0}}{T}\exp\left(-\frac{Q}{kT}\right)$$

where $\sigma_{0}$ is the pre-exponential factor, k represents the Boltzmann constant, and Q denotes the activation energy. The activation energy ($E_{a}$) decreased progressively from 0.988 eV (4Ce4YSZ) to 0.891 eV (4Zn_4Ce4YSZ). These results suggest that the oxygen vacancies formed due to ZnO doping did not form defect associations by interacting with positively charged Zn^2+^.

**Figure 11:**
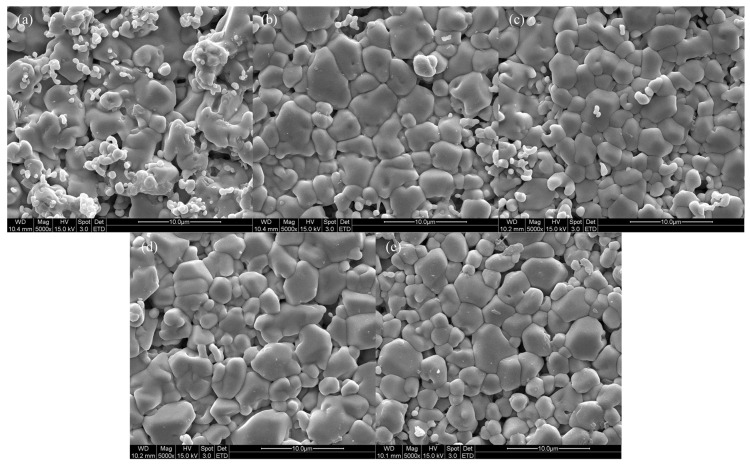
SEM surface images of (**a**) 4Ce4YSZ, (**b**) 0.5Zn_4Ce4YSZ, (**c**) 1Zn_4Ce4YSZ, (**d**) 2Zn_4Ce4YSZ, and (**e**) 4Zn_4Ce4YSZ after molten salt test.

**Figure 12:**
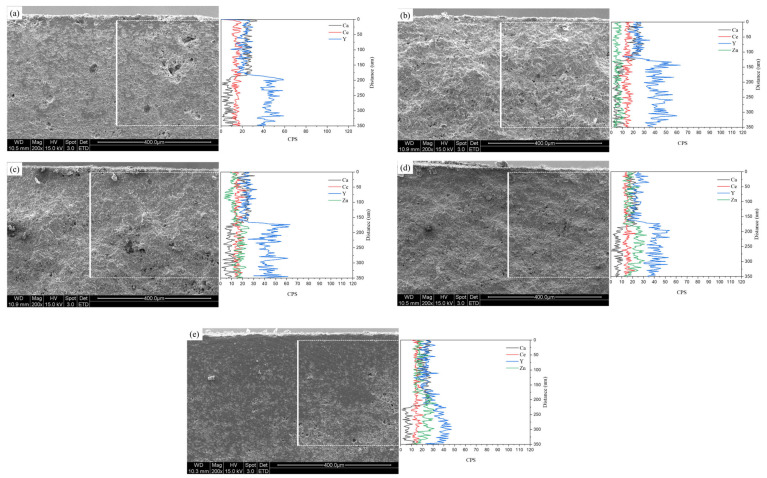
SEM-EDS line scan of (**a**) 4Ce4YSZ, (**b**) 0.5Zn_4Ce4YSZ, (**c**) 1Zn_4Ce4YSZ, (**d**) 2Zn_4Ce4YSZ, and (**e**) 4Zn_4Ce4YSZ after molten salt test.

Figure 11 displays SEM images of the sample surfaces after a 100 h molten salt test at 1000 °C using the eutectic composition of CaF_2_ and NaF. Some remnants of the molten salt can be observed on the sample surface. In all samples, corrosion occurred due to the molten salt, resulting in the formation of pores, and the grains were larger than those before the corrosion. To confirm the destabilization caused by the molten salt, cross-sections were subjected to EDS line scans, as depicted in Figure 12. The detection of the Ca element in the EDS line scan indicates the penetration of the molten salt, and the estimation of the depth of molten salt infiltration was possible to estimate through the decrease in Ca intensity. The molten salt penetration depth decreased from 183.8 μm (4Ce4YSZ) to 128.9 μm (0.5Zn_4Ce4YSZ) and then increased to 218.9 μm (4Zn_4Ce4YSZ). The Y element exhibited low intensity on the surface, which increased almost in correspondence with the depth of salt penetration. This result indicates corrosion by the molten flux and the formation of a yttrium depletion layer (YDL) [36]. A Zn element intensity decrease was observed in 1, 2, and 4Zn_4Ce4YSZ, suggesting the formation of a depletion layer due to corrosion, but the decrease in Zn intensity was lower than that of yttrium. On the other hand, Ce element loss was minimal, indicating the stability of the acidic stabilizing element, CeO_2_, in the SOM electrolysis process [8].

**Figure 13:**
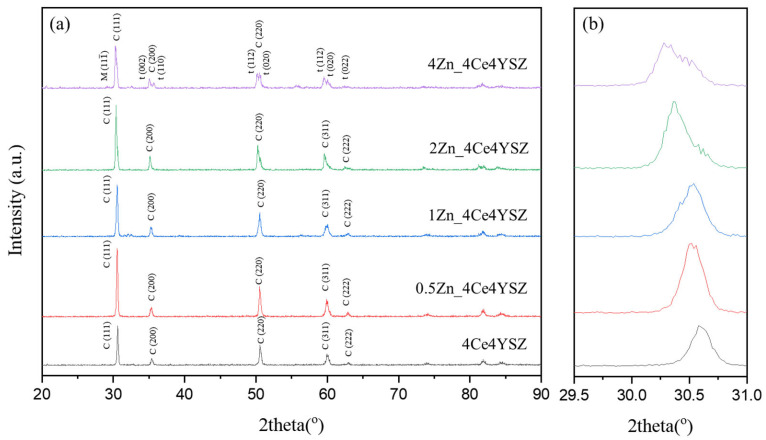
(**a**) XRD diffraction patterns; (**b**) (111) peaks of ZnO-doped 4Ce4YSZ after molten salt test.

Figure 13 shows the XRD of the sample surfaces after the molten salt test, and monoclinic phase formation was observed in the 4Zn_4Ce4YSZ sample, indicating destabilization due to corrosion. The higher-angle shift in the (111) peak observed in all samples after corrosion indicates zirconia lattice contraction due to the expulsion of Y^3+^ (1.04 Å), which was larger than Zr^4+^ (84 Å), from the lattice during corrosion. If Zn^2+^ had leached, there would have been a lower-angle shift, as Zn^2+^ (60 Å), which is smaller than Zr^4+^ (84 Å), exited the zirconia lattice. However, the results show the opposite. These results indicate that yttrium leached more readily than zinc, consistent with the EDS line scan results.

## 4. Conclusions

We doped ZnO to improve the ionic conductivity and sinterability of 4Ce4YSZ and investigated oxygen vacancy ordering and destabilization behavior due to corrosion. The synthesized (4Ce4YSZ)_1−x_ (ZnO)_x_ powders exhibited an increase in the proportion of the cubic phase with increasing ZnO content. Additionally, the higher angle shift of the (111) peak and a reduction in the spacing of the (011)_t_ plane observed in TEM-SAED confirms the substitutional incorporation of smaller Zn^2+^ ions (60 Å) compared to Zr^4+^ (84 Å), leading to lattice contraction. In the analysis of the local atomic structure using EXAFS, an increase in ZnO doping was found to lead to a decrease in Zr-O1 peak intensity, indicating an increase in oxygen vacancies. The rightward shift of the Zr-O1 peak position suggests an increase in the Zr-O1 interatomic distance, indicating that the formed oxygen vacancies moved farther away from the Zr-neighboring oxygen shell. In the Y K-edge Fourier transform data, there was little change in the intensity and distance of the Y-O1 peak, indicating that the formed oxygen vacancies did not occur in the Y neighboring oxygen shell. These results suggest that the formed oxygen vacancies primarily resided in the Zn neighboring oxygen shell rather than in the Y and Zr neighboring oxygen shells. The measured relative density results show that at 1200 °C, 4Ce4YSZ had a relative density of 71.6%, whereas 4Zn_4Ce4YSZ exhibited a relative density of 80.2%, confirming an improvement in the sinterability by ZnO doping. The conductivity, initially at 0.0430 S/cm in 4Ce4YSZ, increased as the ZnO doping level increased, reaching 0.0870 S/cm in 4Zn_4Ce4YSZ. This improvement in conductivity was attributed to the presence of ZnO dissolved in ZrO_2_, leading to the formation of oxygen vacancies and consequently enhancing conductivity. While yttrium leached from the surfaces of all specimens, Ce remained consistently stable at the interfaces. Therefore, it is presumed that CeO_2_, still present as a stabilizer within zirconia, reduced the phase transformation (from cubic and tetragonal to monoclinic). The decrease in the Zn intensity due to corrosion, as observed in the EDS line scan results, was lower than the decrease in the yttrium intensity. Furthermore, after corrosion, all samples exhibited a higher angle shift, indicating that the zinc had better corrosion resistance than the yttrium. The penetration depth of the molten salt decreased from 183.8 μm in 4Ce4YSZ to 128.9 μm in 0.5Zn_4Ce4YSZ, indicating that ZnO doping contributed to improved corrosion inhibition.

## Data Availability

The data and analysis in this study are available on request from the corresponding authors.
